# Biological Sex as a Moderator of Work Determinants of Health: Implications for Work and Stress

**DOI:** 10.3390/healthcare12020135

**Published:** 2024-01-08

**Authors:** Joy L. Hart, Brad Shuck, Jesse Owen, Kandi L. Walker, Rachel J. Keith

**Affiliations:** 1 Department of Communication, College of Arts and Sciences, University of Louisville, Louisville, KY 40292, USA; kandi.walker@louisville.edu; 2 Christina Lee Brown Envirome Institute, School of Medicine, University of Louisville, Louisville, KY 40202, USA; rachel.keith@louisville.edu; 3 Department of Educational Leadership, Evaluation, and Organizational Development, College of Education and Human Development, University of Louisville, Louisville, KY 40292, USA; brad.shuck@louisville.edu; 4 Department of Counseling Psychology, Morgridge College of Education, University of Denver, Denver, CO 80208, USA; jesse.owen@du.edu; 5 Department of Environmental Medicine, School of Medicine, University of Louisville, Louisville, KY 40202, USA

**Keywords:** biomarkers, workplace culture, biological sex, stress, health, catecholamines

## Abstract

This study examined whether biological sex moderates the relationship between experiences of workplace culture and urinary levels of catecholamines and their metabolites. We conducted a series of regression analyses (predictors: 3-methoxytyramine (3MT), 5-hydroxyindolacetic (5HIAA), and dopamine (DA); outcomes: employee engagement and workplace culture) in a sample of 218 participants. Compared to men, women rated workplace culture less positively (*r* = −0.210; *p* < 0.01) and had stronger positive associations with 3MT (*r* = 0.328; *p* < 0.001), DA (*r* = 0.376; *p* < 0.001), and 5HIAA (*r* = 0.168; *p* < 0.01). There was a significant moderation effect between 3MT and sex on employee engagement (b = −1.76 (SE = 0.84); *p* < 0.01), and 3MT had a positive significant association for men with engagement (*p* < 0.05); however, there was no significant association for women. Findings suggest that for women, less positive experiences with workplace culture could elevate 3MT, stimulating sympathetic nervous tone and potentially amplifying risks for negative health outcomes. Conversely, men who reported higher employee engagement had higher levels of 3MT, suggesting possible health risks associated with high levels of engagement, rather than lack of engagement. Overall, study findings suggested differential health risks based on biological sex, potentially impacting health risk policy development.

## 1. Introduction

Although there is considerable interest in the relationship between work experiences and employee health, few studies have examined associations between biological demographics and biomarkers of employee health. For example, stress and the influence of biological demographics at work has been a source of interest [1,2,3,4], and previous research has suggested that biological demographics can influence workplace experiences of stress [5,6]. Those differing experiences can affect a host of outcomes, including organizational performance [7], cardiovascular morbidity [8], and psychosocial risk [6].

Recently, the relationship between workplace culture and stress has been of increasing interest [1,9]. An emerging framework, Work Determinants of Health (WDoH), suggested that social and emotional experiences of work influenced biological outcomes, including an elevated risk for chronic disease [10]. Using WDoH as a primary framework, workplace culture and levels of employee engagement have been identified as important regulators of stress-related sympathetic tone and critical modifiers of cardiovascular disease risk, mental health, diabetes, and obesity using biomarkers as predictors [11]. In early work in this area using urinary levels of catecholamines as biomarkers of sympathetic nervous system activity, participants had lower levels of dopamine and its metabolite, 3-methoxytyramine, when they indicated working in a more positive workplace environment. Indicators signaling shifts in health status, such as a person’s biomarkers, routinely serve as predictors for the risk of chronic health outcomes. Catecholamines, a distinctive biomarker type, are used to examine stress levels by evaluating heightened or diminished responses in the sympathetic nervous system. These responses have been associated with health complications, including chronic diseases [10,11].

At present, biological sex has yet to be examined as a potential moderator within the WDoH work-stress biological framework, despite significant implications for both policy and practice. It seems possible that biological sex could influence the way in which work is both socially and emotionally experienced and, as a result, influence biological response(s). The purpose of this study was to examine the role of biological sex in moderating the relationship between workplace culture and preclinical biomarkers of health. Toward these goals, we explored the following research question:

R_1_: Does an employee’s biological sex moderate the relationship between experiences of workplace culture and urinary levels of catecholamines and their metabolites?

Below, we describe our method, analysis, and findings and then examine the theoretical and practical implications of this work.

## 2. Materials and Methods

### 2.1. Procedures and Participants

Data were collected at two independent points in time. First, participants were recruited to participate in the Health, Environment and Action in Louisville (HEAL) study during 2018–2019. HEAL is an on-going cardiovascular risk cohort focused on risk factors for chronic disease in South Louisville, Kentucky. Biological samples, including clean catch urine samples, were collected at study visits prior to the administration of questionnaires. Post biological sample collection, 733 participants, a sub-sample of the total study population, received an emailed invitation to complete a series of scale-questionnaires to investigate workplace culture. Questionnaires were completed online and asked about stress, depression history, employment history, and employment views. Responses were collected and managed using Research Electronic Data Capture (REDCap) electronic data capture tools hosted at the University of Louisville. Compensation was provided for time. Study-related procedures and measures were approved by the University of Louisville’s Institutional Review Board (IRB# 15.1260 and 19.1047), and informed consent was obtained prior to administering questionnaires and collecting biological samples.

Of those emailed, 243 responded for a participation rate of 33%. Of these responses, 17 were missing half or more of the questionnaire data and were excluded. In total, 8 urine samples were missing; thus, the analytic sample for the current study was 218 participants. The average age of participants was 46 years (SD = 12; range 26–71). The majority were White (82.30%), followed by 13.80% African American/Black and 3.80% Latinx. Additionally, 67.70% identified as women and 32.30% as men.

### 2.2. Measures

#### 2.2.1. Demographic Questions

Participants were invited to provide several demographic indicators including biological sex, ethnicity, and age. Demographic data were used to provide context for the overall distribution of the sample. Biological sex was used as a moderator in later models.

#### 2.2.2. Workplace Culture

Two measures were used to examine overall workplace culture. First, we employed a short measure of workplace culture to explore antecedental conditions of work. Second, because research on both stress and work has focused efforts on engagement, we used employee engagement as an outcome indicator of culture. Research has reliably connected employee engagement as a psychological by-product of antecedental conditions of work, yet the two remain independent in experience and outcome, c.f. [12]. Both scales are described below.

To assess engagement, the Employee Engagement Scale-6 (EES-6) was completed. Questions on the EES-6 were measured on a 5-point Likert scale, with 1 indicating strongly disagree and 5 indicating strongly agree. A sample item from the EES-6 is “*Working at my current job has a great deal of personal meaning for me*”. The EES has demonstrated strong internal consistency (*a* = 0.91) and acceptable model fit (CFI = 0.93; TLI = 0.91; $c_{44}^{2}$ = 741.17, *p* < 0.001) in previous research [12]. Cronbach’s alpha for the EES-6 in the current study was 0.83.

To assess workplace culture, the Cognitive Workplace Appraisal Scale-11 (CWAS-11) was completed. Each item on the CWAS-11 was designed to understand an antecedental dimension of workplace culture using a 5-point Likert scale, with 1 indicating strongly disagree and 5 indicating strongly agree. A sample item from the CWAS-11 is “*I am supported by my supervisor*”. The CWAS-11 has demonstrated strong internal consistency (*a* = 0.87) and acceptable model fit (CFI = 0.99; TLI = 0.99; c251 = 459.89, *p* < 0.001) in previous work [12]. Cronbach’s alpha for the CWAS-11 in the current study was 0.90.

#### 2.2.3. Catecholamine Measures

Urinary levels of catecholamines were measured by UPLC-MS/MS, as previously described [13]. Briefly, 30 μL of urine was thawed on ice, vortexed, and diluted 1:10 with 0.2% formic acid containing isotopic labeled internal standards. The urine was analyzed on an UPLC-MS/MS instrument (ACQUITY UPLC H-Class system and Xevo TQ-S micro triple quadrupole mass spectrometer, all from Waters Inc., Milford, MA, USA). Separation was performed on a Waters Acquity UPLC HSS PFP (150 mm × 2.1 mm, 1.8 μm) column with a binary gradient comprised of 0.2% formic acid (Solvent A) and methanol (Solvent B). Three multiple reaction monitoring (MRM) transitions were set up for each sample: one for quantification, one for confirmation, and one for labeled internal standard. At least 12 data points were collected for each peak. Analytes were quantified using peak area ratio based on 8 point-standard curves run before and after the urine samples. The concentration values of analytes were normalized to urinary creatinine level, which was measured on a COBAS MIRA-plus analyzer (Roche, Branchburg, NJ, USA) with Infinity Creatinine Reagent (Thermo Fisher Scientific, Waltham, MA, USA).

### 2.3. Data Analysis

To examine whether biological sex moderated the association between experiences of workplace culture and urinary levels of catecholamines and their metabolites, we conducted a series of regression analyses. We had two primary outcome variables: (a) employee engagement and (b) workplace culture. The predictors were 3-methoxytyramine (3MT), 5-hydroxyindolacetic (5HIAA), and dopamine (DA). Biological sex was used as the moderator. Analyses were conducted with bootstrapping methods (1000 samples) in SPSS. To normalize the predictor variable, catecholamines were logit transformed prior to analysis. For the regression analyses, multivariate outliers were screened, and all values were within acceptable limits.

## 3. Results

Results demonstrated significant correlations between employee engagement and workplace culture with 3MT (*r* = −0.207; *p* < 0.05 and *r* = −0.308; *p* < 0.001, respectively) and DA (*r* = −0.206; *p* < 0.05 and *r* = −0.228; *p* < 0.01, respectively); however, not with 5HIAA. Compared to men, women rated their workplace culture less positively (*r* = −0.210; *p* < 0.01). Compared to results for men, results for women had stronger positive associations with 3MT (*r* = 0.328; *p* < 0.001), DA (*r* = 0.376; *p* < 0.001), and 5HIAA (*r* = 0.168; *p* < 0.01), respectively.

Table 1 shows the results for moderation tests between participants’ sex and 3MT, DA, and 5HIAA on employee engagement and workplace culture. There was a significant moderation effect between 3MT and participant sex on employee engagement (b = −1.76 (SE = 0.84); *p* < 0.01).

**Figure 1:**
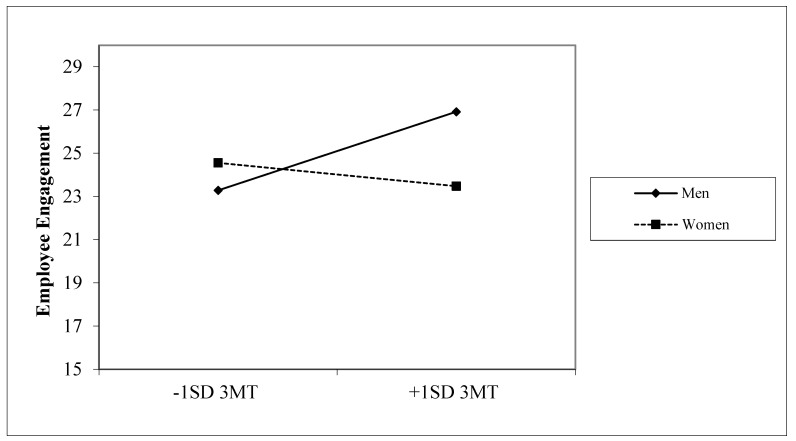
Employee engagement interaction effect. Notes: 3MT = 3-methoxytyramine; SD = standard deviation.

As seen in Figure 1, 3MT had a positive significant association for men with employee engagement (*p* < 0.05); however, there was no significant association for women. There was also a trend for a moderation effect between 3MT and participant sex on workplace culture (b = −2.48 (SE = 1.46); *p* < 0.10). Additionally, there was a trend for a moderation effect for DA and participant sex on workplace culture (b = −2.21 (SE = 1.34); *p* < 0.10). There were no significant moderation effects for 5HIAA.

## 4. Discussion

Previous work has examined gender differences in perceptions of workplace experience, noting disparities between women and men in areas such as stress [4]. However, little research has explored how biological sex differences influence biomarkers of health and risk due to working experiences. Such work should include differences in work experiences specifically and how that risk manifests for employees bounded by biological sex markers.

Specifically, the findings of this study suggest significant relationships between several biomarkers and perceptions of work culture and employee engagement. As expected, work culture and employee engagement were strongly correlated. Further, both work culture and employee engagement were each significantly negatively correlated with 3MT and DA, but not with 5HIAA. These findings indicated that perceptions of the workplace environment are related to key biomarkers, suggesting health risks may differ between biological sex markers based on workplace culture.

Although concerns about the health effects of stress at work are frequent topics in the academic literature as well as in the popular press, little work has examined biomarkers to document health risks. In this study, women assessed their workplace culture less positively than men, and women have a stronger association with catecholamines than men, irrespective of workplace culture. The data suggested that for women, less positive experiences with workplace culture may result in elevated 3MT, stimulating a sympathetic nervous tone which may amplify risks for long-term chronic diseases or other negative health outcomes. Conversely, men who reported higher workplace engagement also had higher levels of 3MT, suggesting that there could be health risks associated with high levels of employee engagement, rather than lack of engagement. Alternatively, given the higher levels of catecholamines in females irrespective of the workplace culture, there could be a blunting of catecholamine release to acute stress that is not seen in males [14]. We note that the absence of similar findings between workplace culture and employee engagement with 5HIAA warrants further exploration in future work. Overall, findings of this study suggest differential health risks based on biological sex, potentially impacting health risk policy development.

Although causality cannot be assessed with the current data, the study findings suggest that a more nuanced approach to examining sex differences in the workplace is needed. For example, perhaps women’s overall experiences of work, through factors such as work culture and employee engagement, lead to different processing and activation of concern factors. In males, gender role stereotypes, being the primary or sole income earner in a household, and the type of occupation may cause a higher level of stress reflected in workplace culture scales and increases in 3MT not seen in overall stress scales. Certainly, a better understanding of such relationships could lead to improvements in workplace conditions and potentially better employee health, especially in these times when employers seek to retain workers.

In this study, women indicated less positive perceptions of work culture than men did. Although much work remains to explicate these relationships, the findings underscore the important role of workplace culture in the employee experience, pointing to the damage that a toxic work culture may inflict and especially the differential negative effects that such a culture may have for women in the workplace, especially in terms of stress responses and the potential for associated long-term health risks.

Several limitations, which may warrant consideration when interpreting findings, should be considered alongside study results. First, the nature of the data prohibits assessing temporality or causality. Second, although these biomarkers provide an important first step in examining potential relationships between work experiences, biomarkers of health, and biological sex differences, the examination of additional biomarkers will be useful in developing a broader understanding of these relationships, especially considering the role of work status or the nature of the work itself. Third, study participants resided in the same geographical region; thus, future assessments with samples representative of larger population areas may yield additional insights and increase the generalizability of study findings. Additionally, we note that our sample size was relatively small, and future research with larger samples may increase generalizability. Fourth, we did not consider participant factors such as wage and work histories, which may have introduced bias. Inclusion of such factors may strengthen future work [15].

## 5. Conclusions

Despite these limitations, this study may pave the way for future research in the area. For example, the study’s findings raise important short-term questions, such as actions that an organization can take to improve workplace culture for women and how these changes may be linked to individual and organizational benefits, and longer-term questions such as concerns regarding workplace insurance and health policies, the ongoing ways that biological sex influences experiences of work and work relationships, and overall influences of work experiences on physical and mental health.

## Figures and Tables

**Table 1:** Results from moderation tests with sex and biomarkers.

**Biological Sex and DA**		
	**Employee Engagement** ***b (se)***	**Workplace Culture** ***b (se)***
Intercept	24.20 (0.69)	46.51 (0.91)
Biological Sex	−0.45 (0.80)	−2.85 (1.18) **
DA	−0.11 (0.71)	0.48 (1.08)
Biological Sex × DA	−0.85 (0.82)	−2.21 (1.34) ~
*R*^2^ (*R*2-*change*)	0.04 (0.01)	0.07 (0.02)
**Biological Sex and 3MT**		
	**Employee Engagement** ***b (se)***	**Workplace Culture** ***b (se)***
Intercept	24.56 (0.67)	46.32 (1.16)
Biological Sex	−0.81 (0.78)	−2.57 (1.36) *
3MT	0.64 (0.74)	0.13 (1.30)
Biological Sex × 3MT	−1.76 (0.84) **	−2.48 (1.46) ~
*R*^2^ (*R*2-*change*)	0.04 (0.03)	0.11 (0.02)
**Biological Sex and 5HIAA**		
	**Employee Engagement** ***b (se)***	**Workplace Culture** ***b (se)***
Intercept	24.70 (0.64)	46.93 (1.18)
Biological Sex	−1.30 (0.76) ~	−3.97 (1.32) **
5HIAA	1.15 (0.71) ~	1.76 (1.24)
Biological Sex × 5HIAA	−0.65 (0.81)	−0.36 (1.42)
*R*^2^ (*R*2-*change*)	0.04 (0.01)	0.09 (0.001)

Notes: ~ *p* < 0.10, * *p* < 0.05, ** *p* < 0.01; Sex: 1 = women, 0 = men; DA = dopamine, 3MT = 3-methoxytyramine, 5HIAA = 5-hydroxyindolacetic.

## Data Availability

The data presented in this study are available on request from the corresponding author. The data are not publicly available due to privacy issues.
